# The effect of institutional learning curve on paediatric ventricular shunt survival: a retrospective cohort study from a new paediatric neurosurgical centre

**DOI:** 10.1007/s00381-025-06927-w

**Published:** 2025-08-18

**Authors:** Michael J. Stuart, Soe Htike, Annabelle M. Harbison, Timothy Ruder, Amelia J. Jardim, Norman Ma, Liam G. Coulthard, Robert A. J. Campbell

**Affiliations:** 1 Department of Neurosurgery, Queensland Children’s Hospital, Brisbane, QLD Australia; 2 Faculty of Medicine, University of Queensland, Brisbane, QLD Australia; 3 Department of Neurosurgery, Townsville University Hospital, Townsville, QLD Australia

**Keywords:** Hydrocephalus, Nursing, Cerebrospinal fluid, Ventriculoperitoneal

## Abstract

**Introduction:**

Ventricular shunt procedures and their outcomes are commonly considered a benchmark for the performance of a neurosurgical service. The influence of an institutional learning curve on the outcomes of neurosurgical procedures has not been established; however, it is important when considering the establishment of neurosurgical centres or the introduction of new operating room teams. A benchmark high-volume procedure such as paediatric ventricular shunt insertion represents an ideal model to study this phenomenon.

**Methods:**

We conducted a retrospective review of a prospectively maintained surgical database, covering 10 years of consecutive cases from a new quaternary Australian paediatric neurosurgical centre. Patients undergoing insertion of a new ventricular shunt were included, while all cases of shunt revision were excluded. The 10-year study period was divided into four eras of 2.5-year duration for analysis.

**Results:**

A total of 427 new ventricular shunt systems were inserted in the initial 10 years since the hospital’s commissioning. There was a significant improvement in overall time to shunt revision from the first era to the second, however no further change thereafter (median overall shunt survival 392 days vs not reached in other eras, *p* < 0.001). Similarly, time in the operating room improved from the first to the second era and reached a steady state thereafter (mean 129 ± 46 min in years 0–2.5 and 103 ± 44 min in years 2.5–5 (*p* < 0.001)).

**Conclusion:**

The institutional learning curve for new ventriculoperitoneal shunt placement appears to plateau within 2.5 years of commissioning a new paediatric neurosurgical hospital.

## Introduction

The insertion of a new ventriculoperitoneal shunt is one of the most common operations in paediatric neurosurgery and is considered a ‘benchmark’ procedure within the specialty [1]. Complications of the initial shunt procedure and subsequent revision are common and highly significant to the long-term outcome of these patients [2, 3]. There is therefore great interest in optimizing care for these vulnerable patients [2–5]. One such strategy is the centralization of healthcare resources into quaternary paediatric specialist hospitals to maximize case volume and institutional experience in managing patients such as these. Although individual surgeons may be experienced, the introduction of new facilities and staff (such as operating room teams) into the management of a complex patient population may require a form of institutional learning curve in order to achieve optimal performance. While the role of individual surgeon case volume and learning curve is well recognized for many procedures [6, 7], less emphasis is often placed on any influence the institutional learning curve may have on clinical outcomes although this effect may be significant [8, 9]. When assessing an outcome such as time to shunt revision (or ‘shunt survival’), many factors may exert an influence—from unfamiliarity with staff or facilities in the operating room to staff discomfort with assessing or observing these patients in the emergency room or wards. Many of these factors are outside the direct control of the treating surgeon, and the potential influence of such factors on shunt survival has not previously been reported.

Opportunities to assess the institutional learning curve for paediatric ventricular shunt insertion in a new hospital are relatively rare occurrences. The description of such a learning curve is of importance to policy decisions by providing benchmarking data relevant to new institutions. For example, this should be considered when interpreting National Surgical Quality Improvement Program (NSQIP) data originating from those facilities. Additionally, the institutional learning curve is a relevant factor to consider when determining when or whether to include an institution in a large multicentre trial or research collaborative. Finally, the major enforced alterations to the surgical milieu engendered by such a change provide an extreme model to study the effect of smaller changes, which are a far more frequent irritant to surgeons, for example, changes in usual operating room staff.

Our centre was commissioned 10 years ago to serve as the sole quaternary paediatric neurosurgical hospital for a population of 5.3 million (1.1 million children) in the State of Queensland, Australia [10]. This centre was formed by the merger of a number of smaller paediatric hospitals and included both the employment of experienced staff from those centres but also the recruitment of a large proportion of new staff in all departments including operating room teams. This represents a rare opportunity to describe the institutional learning curve for new paediatric ventricular shunt insertion and shunt survival in such a setting.

The aims of this study were to assess the changes in shunt survival and operating room times over the first 10 years of a new paediatric hospital to describe the institutional learning curve.

## Methods

**Fig. 1:**
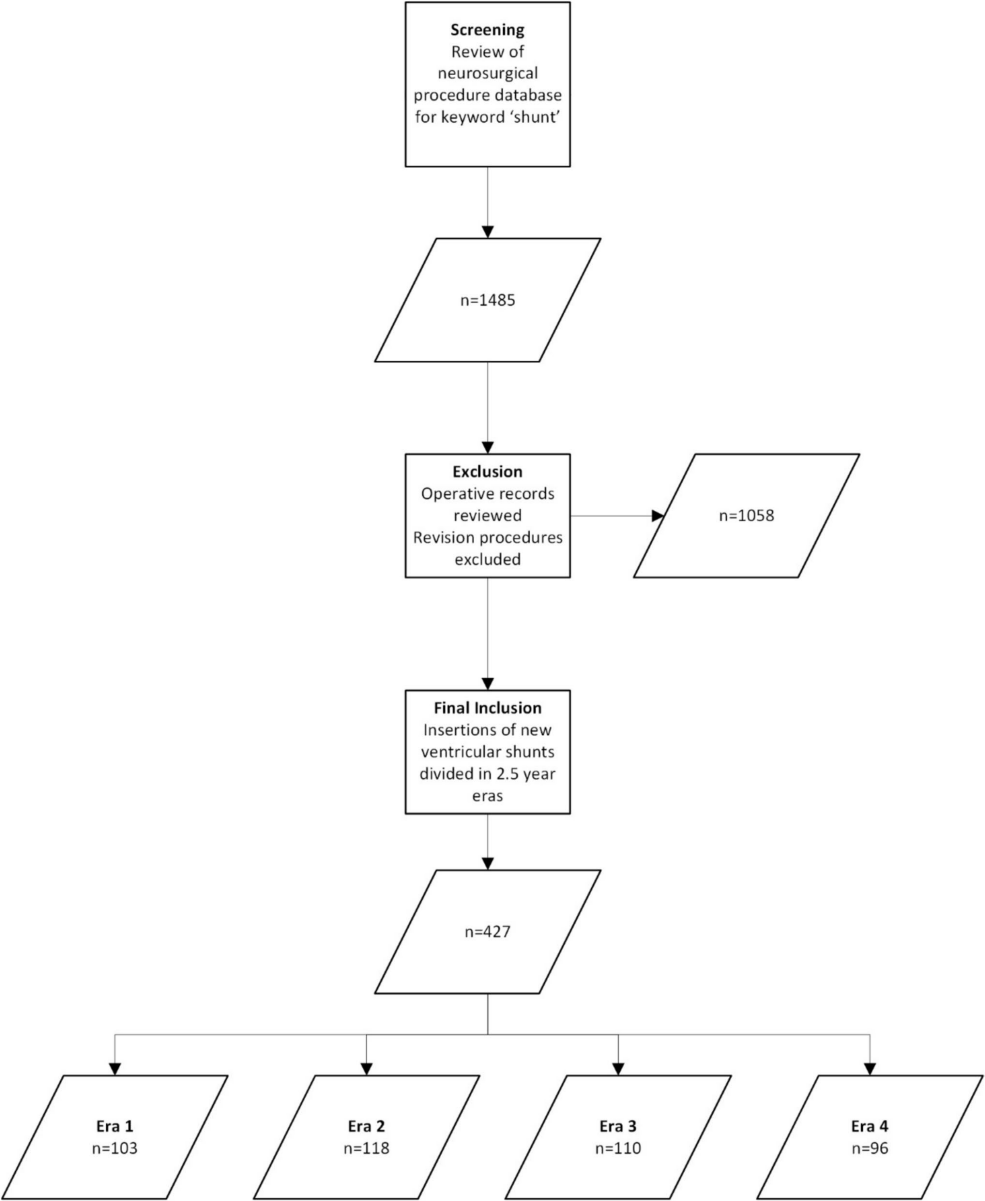
Case inclusion flowchart

A retrospective review of a prospectively maintained database was conducted, including all patients who underwent cerebrospinal fluid diversion procedures at the Queensland Children’s Hospital from 29 November 2014 to 1 September 2024. All cases undergoing insertion of an entire new ventricular to peritoneal, pleural, or atrial shunt were included. Cases of converting an existing ventricular reservoir device into a shunt system were included. Cases of non-ventricular proximal catheter placement such as subdural, cyst, or lumbar placement were excluded. Aside from the previously indicated ventricular reservoir, all cases of revision surgery where any part of a previously existing shunt system was retained were also excluded. Second or further revisions were not included in the revision as the survival of a revised system may be dependent on time since last revision and not equivalent to the survival of a new shunt system [11] (Fig. 1).

‘Eras’ for ventriculoperitoneal shunt insertion were defined in 2.5-year intervals and numbered 1–4 in chronological order.

Procedures were performed with the use of neuronavigation, antibiotic impregnated catheters, and mini-laparotomy for abdominal access as standard of care. From the commissioning of the hospital to the end of the data capture period, infection prevention was carried out according to the protocol described by the Hydrocephalus Clinical Research Network [12].

We reviewed case records and imaging from index procedure and any subsequent shunt revision procedures performed across Queensland using statewide electronic medical records. Data entry for all cases undergoing revision was reviewed in duplicate by two neurosurgeons and any conflicts resolved by consensus.

The primary outcome was defined as overall shunt survival or time to shunt revision over the eras, while time in the operating room was defined as the secondary outcome. ‘Shunt-related mortality’ was defined to exclude palliation related to malignancies, even if a shunt was in situ. Deaths due to intended palliation in the setting of progressive hydrocephalus, with confirmed hydrocephalus on neuroimaging or out of hospital cardiac arrest in a patient with a shunt, were included as ‘shunt-related mortality’. Shunt-related mortality was included in the time to shunt revision survival analysis as ‘shunt failures’.

Distribution of continuous data was assessed for normality using Shapiro–Wilk W test. For comparisons across the four eras, the independent-samples Kruskal–Wallis test or ANOVA were used depending on the distribution of the data. Similarly, where two groups were compared, Student’s *T*-test was used for continuous data conforming to a normal distribution while the Mann–Whitney *U* test was utilized where it was not. Univariate analysis of categorical variables employed Pearson Chi-squared test. Analysis of time to shunt revision was undertaken with Kaplan–Meier survival curves and analysis by both Wilcoxon (to detect early differences) and log-rank (to detect later differences). Multivariate analysis was conducted in a Cox proportional hazard model including all factors found to be significant on univariate analysis. Cases with missing data were excluded casewise from multivariate analysis. Multivariate results are presented as hazard ratios (HR) with 95% confidence intervals (95% CI). Repeated revisions within 1 year were compared across all eras pairwise by the SPSS independent samples medians test. Statistical significance was set at *p* < 0.05. All data analysis was performed using SPSS 30 (IBM Corporation, New York, 2024).

Ethical approval was obtained from the Queensland Children’s Hospital human research ethics committee (HREC/24/QCHQ/108644).

## Results

Over the study period of 10 years, 1485 shunt procedures were performed at the Queensland Children’s Hospital, of which 427 involved the placement of a new ventricular-peritoneal shunt system (Fig. 1). The demographics of this sample have been recently reported [5, 13]. In brief, 190 (44%) patients were female with a mean age of 5.2 years (range premature, 18 years). The most common causes of hydrocephalus were tumour (34%), congenital (including Chiari II) (29%), and intracerebral haemorrhage (24%) (Table 1). Most cases (413/427, 97%) were ventricular-peritoneal shunts and there were 12 (2.8%) cases of ventricular-atrial shunts. Most proximal catheters were inserted into the right lateral ventricle (79%), 19% into the left lateral ventricle, and four into the 4th ventricle (< 1%). One hundred seventy-three of the 427 (41%) shunts underwent revision during the period of follow-up, at a median of 103 days (range 0–2942 days). The median follow-up period was 4.85 years (range 0–10 years). Median overall shunt survival (or median time to first shunt revision) was 8.1 years. The most common indications for shunt revision were proximal catheter obstruction (51/173, 29%) followed by infection (29/173, 17% of revisions) and valve blockage (23/173, 13%). The overall shunt infection rate was 29/427 (7%). The indications for revision did not change across the defined eras. Of particular importance, the rate of shunt infection did not change (6% era 1, 9% era 2, 5% era 3, 7% era 4; *p* = 0.67) (Table 2).

**Table 1:** Demographics and median shunt survival of entire cohort

Variable	Era 1
Age (mean + range, years)	5.2 (premature, 18)
Sex (percent female)	190 (45%)
Aetiology of hydrocephalus	
Congenital (including Chiari II)	124 (29%)
Haemorrhage	102 (24%)
Tumour	144 (34%)
Idiopathic Intracranial hypertension	13 (3%)
Infection	24 (5.6%)
Other	20 (5%)
ASA score	
1	8 (2%)
2	111 (26%)
3	275 (64%)
4	33 (8%)
Time in theatre (mean ± standard deviation, minutes)	101 ± 44
Side	
Right	337 (79%)
Left	82 (19%)
Bilateral	4 (1%)
4th ventricle	4 (1%)
Proximal site	
Parietal-occipital	365 (86%)
Frontal	55 (13%)
Other	7 (2%)
Distal site	
Peritoneal	413 (97%)
Atrial	12 (3%)
Pleural	1 (1%)
Other	1 (1%)
Valve	
Delta	261 (61%)
Certas	74 (17%)
OSV	58 (14%)
Polaris	15 (4%)
Strata	15 (3.5%)
Other fixed	4 (1%)
Other programmable	1 (1%)
Antisiphon device	292 (64%)
Flow regulated valve	58 (14%)
CSF protein > 300 mg/L	122 (29%)
Shunt Infections	27 (6%)
Median shunt survival	NR
Total	

Percentages presented are in proportion to the number of cases complete data for that datapoint. *NR* not reached. *ASA* American Society of Anaesthesiology score, *CSF* cerebrospinal fluid, *OSV* Orbis-Sigma Valve

**Table 2:** Demographics and median shunt survival by era

Variable	Era 1	Era 2	Era 3	Era 4	*p* value
Age (Mean + range, years)	5.1 (0–18)	5.5 (0–18)	5.0 (0–18)	5.0 (0–18)	0.91
Sex (percent female)	40 (39%)	64 (54%)	44 (40%)	42 (44%)	0.08
Aetiology of hydrocephalus					0.23
Congenital (including Chiari II)	37 (36%)	26 (22%0	34 (31%)	27 (28%)	
Haemorrhage	23 (22%)	35 (30%)	24 (22%)	20 (21%)	
Tumour	34 (33%)	40 (34%)	39 (36%)	31 (32%)	
Idiopathic Intracranial hypertension	1 (1%)	5 (4%)	3 (3%)	4 (4%)	
Infection	1 (1%)	8 (7%)	5 (5%)	10 (10%)	
Other	7 (7%)	4 (3%)	5 (5%)	4 (4%)	
ASA score					0.14
1	3 (3%)	0	1 (1%)	4 (4%)	
2	26 (25%)	41 (35%)	24 (22%)	20 (21%)	
3	68 (66%)	67 (557%)	76 (69%)	64 (67%)	
4	6 (6%)	10 (9%)	9 (8%)	8 (8%)	
Time in theatre (mean ± standard deviation, minutes)	129 (± 46)	103 (± 44)	85 (± 36)	87 (± 34)	< 0.001
Side					0.41
Right	78 (76%)	92 (78%)	88 (80%)	79 (82%)	
Left	21 (20%)	25 (21%)	20 (18%)	16 (17%)	
Bilateral	3 (3%)	1 (1%)	0	0	
4th ventricle	1 (1%)	0	2 (2%)	1 (1%)	
Proximal site					< 0.001
Parietal-occipital	80 (78%)	107 (91%)	96 (87%)	82 (85%)	
Frontal	23 (22%)	8 (7%)	13 (12%)	11 (12%)	
Other	0	1 (1%)	1 (1%)	3 (3%)	
Distal site					0.90
Peritoneal	102 (99%)	112 (95%)	109 (99%)	90 (94%)	
Atrial	1 (1%)	5 (4%)	0	6 (6%)	
Pleural	0	1 (1%)	0	0	
Other	0	0	1 (1%)	0	
Valve					< 0.001
Delta	52 (51%)	71 (60%)	74 (67%)	64 (67%)	
Certas	5 (5%)	16 (14%)	24 (22%)	29 (30%)	
OSV	33 (32%)	22 (19%)	2 (2%)	1 (1%)	
Polaris	4 (4%)	4 (4%)	7 (6%)	0	
Strata	9 (9%)	4 (3%)	0	1 (1%)	
Other fixed	0	1 (1%)	2 (2%)	1 (1%)	
Other programmable	0	0	1 (1%)	0	
Antisiphon device or flow regulated valve present	86 (84%)	98 (83%)	82 (75%)	84 (88%)	0.24
CSF protein > 300 mg/L	52 (59%)	36 (53%)	19 (46%)	15 (42%)	0.27
Shunt Infections	6 (6%)	10 (9%)	6 (5%)	7 (7%)	0.67
Median shunt survival (days, 95% CI)	392 (44–740)	NR	NR	NR	< 0.001
Revisions within one year of index shunt					< 0.001
1	27 (26%)	19 (16%)	21 (19%)	18 (19%)	
2	16 (16%)	11 (9%)	5 (5%)	2 (2%)	
3	4 (4%)	1 (1%)	1 (1%)	1 (1%)	
4 +	4 (4%)	4 (3%)	0	0	
Total	103	118	110	96	427

Percentages presented are in proportion to the number of cases complete data for that datapoint. *NR* not reached. *ASA* American Society of Anaesthesiology score, *CSF* cerebrospinal fluid, *OSV* Orbis-Sigma Valve

There were no significant demographic differences between the defined eras; however, there were several evolutions in technique over time. The early eras had an increased proportion of frontal shunt approaches (22% era 1, 7% era 2, 12% era 3, 12% era 4; *p* < 0.001) and the use of Orbis-Sigma valves (OSV) (32% era 1, 19% era 2, 2% era 3, 1% era 4; *p* < 0.001) while practice shifted to prefer the use of a Codman Certas programmable valve (5% era 1, 14% era 2, 22% era 3, 30% era 4; *p* < 0.001) and a parietal-occipital approach (78% era 1, 91% era 2, 87% era 3, 85% era 4; *p* < 0.001) in later eras. There was a corresponding shift from the use of flow-regulated valves to anti-siphon devices, with figures corresponding to the prevalence of OSV valves in each era (*p* < 0.001) (Table 2).

At the time of commissioning of the Queensland Children’s Hospital, all neurosurgeons were well advanced in their personal learning curves, with a mean of 23 years (range 11–33) practice in paediatric neurosurgery.

**Fig. 2:**
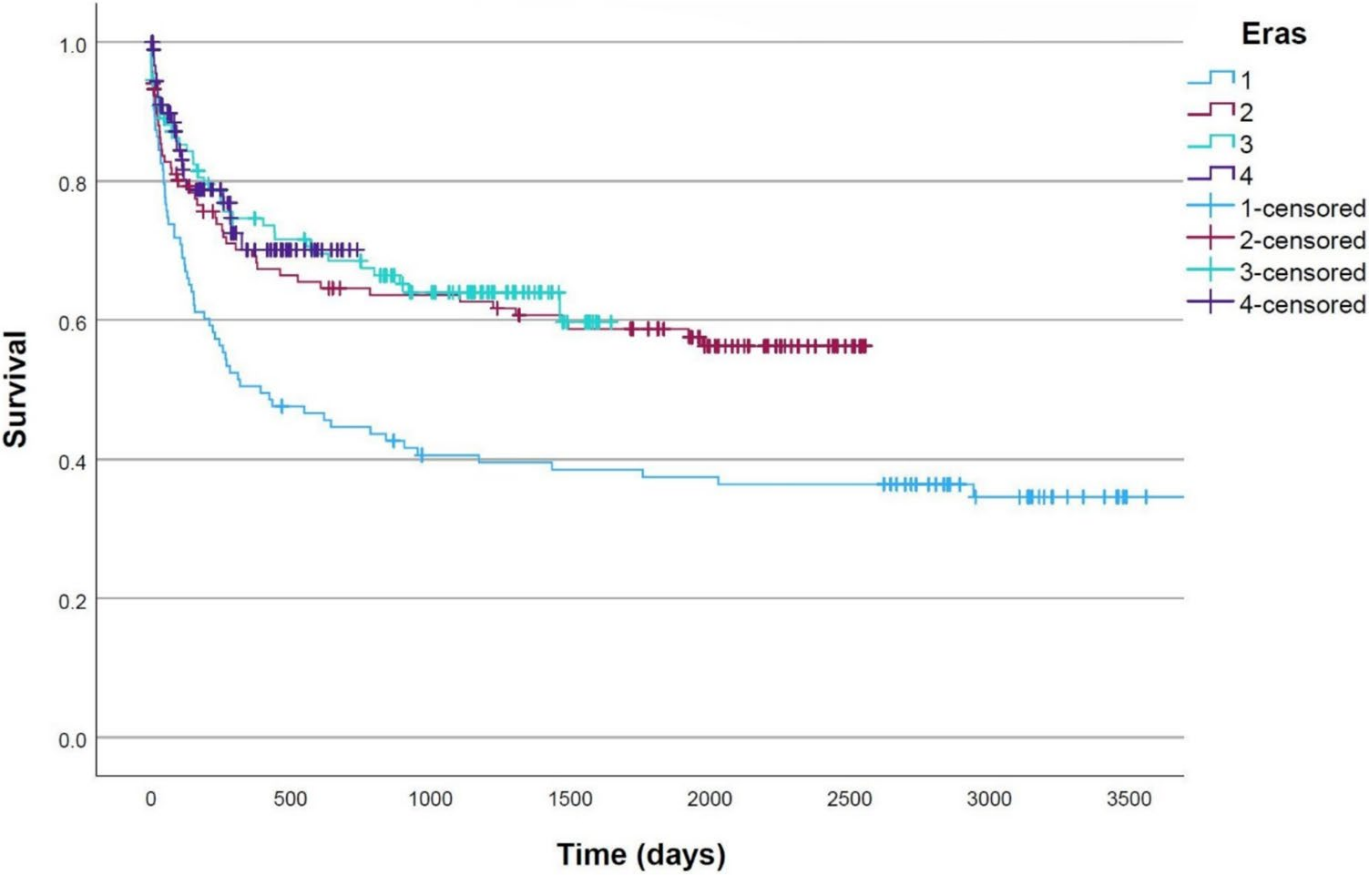
Kaplan–Meier survival curve of time to shunt revision after index shunt insertion. Significantly worse with median shunt survival 392 days in era 1 and not reached in other eras (*p* < 0.001)

Kaplan–Meier analysis of overall shunt survival (or time to shunt revision) demonstrated a statistically significant improvement after era 1 (median shunt survival 392 days vs not reached in other eras, *p* < 0.001); however, the curves remained consistent thereafter (Fig. 2). Due to the shorter follow-up (and accordingly lower number of revision events) in later eras, this analysis was repeated with comparison of era 1 to other eras combined and remained statistically significant.

**Fig. 3:**
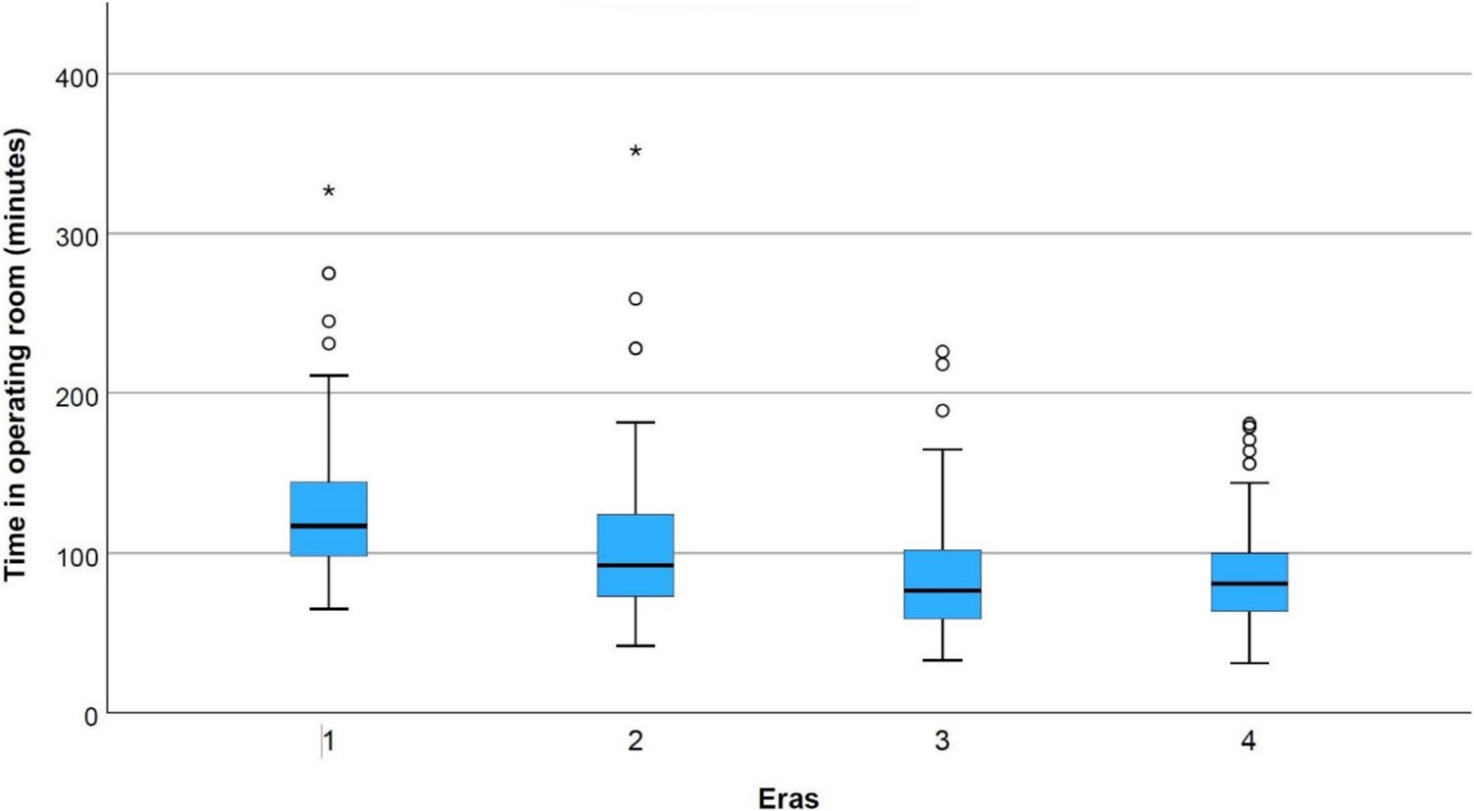
Time in operating room by era. * indicates statistically significant difference

On univariate analysis, time in the operating theatre significantly declined from a mean of 129 ± 46 min in era 1 to 103 ± 44 in era 2 (*p* < 0.001). Ongoing but not statistically significant improvements were achieved in eras 3 and 4 (Table 2 and Fig. 3).

A multivariate Cox proportional hazard model was utilised, including the era along with all factors demonstrated to be significantly associated with shunt failure in a previously published analysis of this cohort [5]. This demonstrated age (HR 0.92, 95% CI 0.89–0.96, *p* < 0.001), time in operating room (HR 1.005, 95% CI 1.001–1.010, *p* = 0.02), and elevated CSF protein (HR 1.66, 95% CI 1.11–2.46, *p* = 0.01), but not era (*p* = 0.2) to be independently associated with time to shunt failure (Table 3). The analysis was also repeated with the first era considered a binary variable compared to the other eras (2–4) combined, and the statistical significance of each variable remained unchanged. Due to the possibility of the 4th era having shorter follow-up, the analysis was repeated with the omission of that era, and findings again remained unchanged.

**Table 3:** Cox proportional hazard regression model

Variable	Hazard ratio	95% confidence interval	Significance (*p* value)
Age	0.92	0.89–0.96	< 0.001
Time in operating theatre	1.005	1.001–1.010	0.02
CSF protein > 300 mg/L	1.66	1.11–2.46	0.01
Era			0.2
1	Reference	Reference	
2	0.78	0.49–1.24	
3	0.67	0.37–1.21	
4	0.46	0.21–1.00	

*CSF* cerebrospinal fluid

**Fig. 4:**
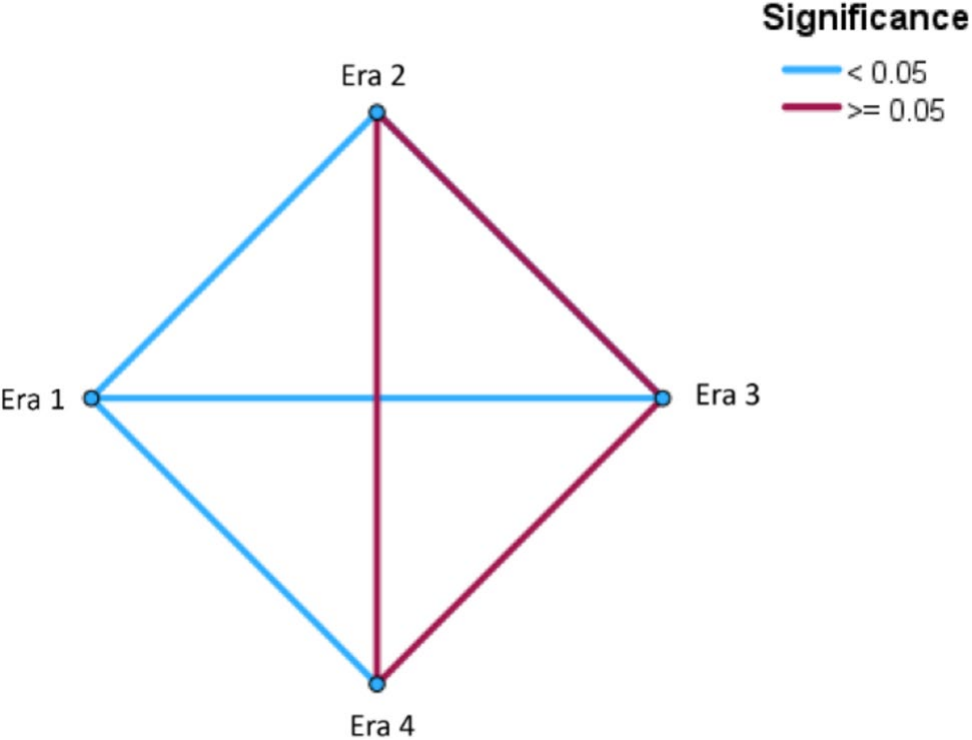
Pairwise comparison between frequencies of shunt revisions within first year of index insertion. Significant differences are only present when comparing era 1 to others

An additional analysis of repeated shunt revisions occurring within 1 year of the index shunt insertion was performed. In the first era, 51/103 (49.5%) of shunts were revised at least once within the first year of insertion in comparison to 35/118 (29.7%) in the second, 27/110 (24.5%) in the third, and 21/96 (21.9%) in the fourth era. The number of repeated shunt revisions within 1 year in the first era was also significantly higher when compared to each other era (era 1 vs 2; *p* = 0.003, era 1 vs 3; 50% vs *p* < 0.001, era 1 vs 4; *p* < 0.001); however, there were no significant differences between eras 2–4 when compared to each other (all *p* > 0.05) (Table 2 and Fig. 4).

## Discussion

This study presents the first description of the institutional learning curve for paediatric ventricular shunt insertion at a new hospital. We found a significantly higher risk of shunt failure or revision within the first 2.5-year era, after which time the failure rate stabilised (Fig. 2). Additionally, shunts inserted in the first 2.5-year era were more likely to undergo repeated revisions within the first year after insertion (Fig. 4). The time in the operating room was also significantly longer in the first 2.5-year era before decreasing in later eras (Fig. 3). Multivariate analysis demonstrated that the time in the operating room, not the ‘era’ itself, was the key independent predictor of early shunt failure. This suggests that factors related to institutional inexperience, such as staff or facility unfamiliarity, contributed to the longer procedures and consequently higher failure rates. On multivariate analysis, the decrease in operating room time appears to largely mediate the observed effect; however, it is impossible to determine the exact cause of this improvement—whether that relates to improvement on the part of operating room staff or surgeons themselves. On the basis of these metrics, it could be asserted that the institutional learning curve appears to plateau within the first 2.5 years of commissioning a new paediatric hospital, and after that time, this factor should not complicate the interpretation of outcomes data for academic or clinical governance purposes.

The potential mechanisms of this decrease in shunt survival require consideration. There did not appear to be a change in the indication for shunt revision over the eras and, importantly, no demonstrable increased burden of infection in the initial period—however, this result should be interpreted with caution. Despite the large sample size, the event rate is low, which results in the study being underpowered to detect such a difference. Shunt obstruction may, however, coexist with subclinical infection, especially with low virulence organisms such as *Cutibacterium acnes*. Our lab does not currently employ routine sonication of explanted shunt components, which may result in a lower culture yield of such subclinical infections [14]. Other mechanisms by which a prolonged operative time may contribute to earlier shunt failure include inadvertent ingress of blood or debris into shunt valves or tubing or indelicate handling of materials, resulting in propensity to fracture or disconnection. Given the absence of change in the proportions of shunt obstruction, fracture, disconnection, or infection over the eras, it is not possible to better specify the mechanism.

The major strengths of this dataset are the comprehensive population-level dataset with strong follow-up due to statewide electronic medical records. There is also relative demographic similarity across the defined eras which allows for meaningful comparison. The influence of any potential surgeon or trainee-level learning curve is minimized due to the seniority of the neurosurgeons at the time of commissioning of the hospital (mean 23, range 11–33), years of paediatric neurosurgical practice, and frequent turnover of trainees (each 6 months) which ensures each 2.5-year era is not heavily influenced by any single operator. Additionally, those differences in technique noted between the early era and later eras, specifically the choice of valve and frontal or parietal/occipital approach, have previously been well described in this dataset and others to have no significant association with shunt failure [5, 15].

Although the ‘sample size’ is population level, the division of that sample into four eras does decrease the event rate of shunt failures in each group. This is particularly relevant in the most contemporary era where a brief follow-up period reduces the event rate further. To overcome this limitation, it would be reasonable to repeat the analysis in 5 to 7 years from the end of the final era after all groups had reached the expected median shunt survival. Additional limitations common to the retrospective design of this study are also relevant to consider. These include limitations in interpreting the incidence of shunt revisions as incidence of shunt failure. The documentation of intraoperative findings is often performed by junior neurosurgical staff and does not always allow certainty regarding the clinical impression of whether true shunt failure was encountered at the time of operation; conservatively, these were all included as shunt failures. That determination may confound the results if the rate of ‘negative shunt exploration’ (where all components were found to be functional) declined over time as a result of increasing surgeon experience; however, due to the retrospective nature of the data capture, this effect is impossible to exclude.

The population-level sample includes all major pathologies which lead to paediatric ventricular shunt placement in a developed nation, but has a notable absence of other pathologies such as tuberculous meningitis. This data is therefore likely to be generalizable to other developed nations only. A further consideration is the absence of any junior neurosurgeons in the department at the time of commissioning of the hospital; however, in the presence of less experienced surgeons to guide the development of policies and protocols, the institutional learning curve may be prolonged.

The insertion of a ventriculoperitoneal shunt is a technically simple procedure in comparison to many neurosurgical procedures. However, when considering the institutional learning curve, the surgeon’s individual learning curve is a relatively small component of the greater whole. Other contributors such as the comfort of the operating room teams with preoperative workflows including positioning/registration of neuro-navigation, intraoperative workflows, and postoperative care are likely to be relatively comparable with other common neurosurgical procedures. In this regard, the plateau for time in the operating room after 2.5 years is likely to be generalizable to many other neurosurgical procedures. This may also be a relevant consideration when introducing a new neurosurgical procedure into an already established centre, or when an institution enforces a change in a neurosurgeon’s regular operating room staff. This study provides indirect evidence that if a novel procedure (or new surgical team) was to be introduced, it could be hoped that steady-state performance was within a similarly brief timeframe.

## Conclusion

The institutional learning curve for new ventriculoperitoneal shunt placement appears to plateau within the first 2.5 years of commissioning a new paediatric neurosurgical hospital. This may be a relevant benchmark for the introduction of new neurosurgical procedures or surgical teams.

## Data Availability

No datasets were generated or analysed during the current study.
